# Progress in Surgery of the Pancreas and Spleen

**Published:** 1902-07-12

**Authors:** 


### Progress in Surgery of the Pancreas and Spleen.

**Pancreatic Cysts** are classified by Moyniham[1] as follows: (1) Retention cysts; (2) Proliferation cysts (*a*) Cystic adenoma, (*b*) Cystic epithelioma; (3) Hydatid cysts; (4) Congenital cystic disease; (5) Hæmorrhagic cysts; Pseudo-cysts. The signs and symptoms warranting a diagnosis of pancreatic cyst are as follow: A patient ill with indefinite symptoms of epigastric uneasiness, weight, pain, and occasional vomiting, begins to lose weight, and on examination a swelling of the upper part of the abdomen is discovered; or a patient suffers an injury of some severity to the upper part of the abdomen, and within a brief period of days or weeks a tumour forms, with or without symptoms, in the epigastric region. As the tumour enlarges, the symptoms become more acute, or, less commonly, remain stationary. Jaundice, intestinal hæmorrhages, or diarrhœa may be observed. The tumour which forms lies generally in the epigastric region, with its most prominent aspect at or near the middle line or between the mid-line and the left costal margin. The tumour is rounded, generally smooth, elastic, or fluctuating, and it may vary in size from time to time. It is dull on percussion in the centre, but above percussion demonstrates the stomach resonance and below that due to the transverse colon. Inflation of these viscera increases the area of their tympany and lessens the central dull area of the tumour. The tumour is fixed; the skin over it, smooth and unwrinkled. In the urine sugar may be found; in the fæces an excess of fat, and of undigested muscle fibre. Exploratory puncture, for purposes of diagnosis is too risky a procedure for the small amount of information it affords. The differential diagnosis of pancreatic cyst may be a matter of insurmountable difficulty. The following are the chief conditions from which pancreatic cysts must be distinguished:—1. Ovarian cystoma. 2. Cysts of the liver. 3. Distended gall-bladder. 4. Cyst of the supra-renal capsule. 5. Fluid tumours of the kidney. 6. Mesenteric cysts. 7. Omental cysts. 8. Cysts in the posterior wall of the stomach. 9. Cysts of the spleen. 10. Retroperitoneal cysts. The operative treatment of pancreatic cysts is limited to three methods:—1. Aspiration. 2. Evacuation and drainage, the cyst being stitched to the abdominal wall. 3. Extirpation, partial or complete. Of these, the second method is the safest and most successful. Heaton[2] records a case of partial excision of the pancreas for multilocular cystic tumour.

**Wounds of the Pancreas.**—Ninni[3] maintains:—1. That punctured or incised wounds of the pancreas should be sutured; if the duct is cut, suture as in intestine, leaving no thread in the lumen, so as to avoid risk of calculi forming. 2. In gunshot wounds, if there is not much laceration, suture in bulk; if lacerated, resect; if the laceration is total one can only plug, for removal of the whole gland is difficult and fatal. 3. The same rules apply to contused wounds. 4. In rupture, there being usually not much laceration, suture. In all cases, if suture does not stop the hæmorrhage, ligature.

**Movable Spleen.**—Wertheim[4] relates a case in which he detected a very tender mass of the size of two fists firmly incarcerated in the pelvic cavity, but in putting the patient under an anæsthetic it slipped up into the abdominal cavity on pressure from below. An exploratory incision was made, and the tumour proved to be the spleen enlarged to about three times the normal size. The pedicle was long and easily secured, the patient making a good recovery from the operation. Hansy[5] relates the case of a patient who, on the fourth day of the puerperium, was seized with a stabbing pain in the left side. A firm, not tender, mass was detected on the right, not the left, side of the abdomen inferiorly. It was of the size of a child's head, and could be pushed into any region of the abdomen. The abdominal walls were thin, and the splenic notch was easily felt. There was no history of malaria and no evidence of leukæmia. The enlarged spleen was removed; the pedicle, which was twisted 180°, was moderately thick, and was tied in segments. The splenic tissues showed pure hyperplasia. The patient was quite well in eighteen days, and the blood showed no change since the operation except a slight diminution in the proportion of leucocytes.

1 Med. Chron., Jan. 1901, p. 241. 2 Brit. Med. Jour., Oct. 19, 1901. p. 1160. 3 Brit. Med. Jour., June 8, 1901. Epitome No. 376. 4 Brit. Med. Jour., Feb. 8, 1902, p. 350. 5 Ibid.

